# Evaluating the current landscape of clinical trials registration and results reporting policies, procedures and staffing at US-based academic centers: Survey revisited

**DOI:** 10.1017/cts.2025.10171

**Published:** 2025-10-13

**Authors:** Anthony Keyes, Jesse Reynolds, Jillian Barron, Sarah White

**Affiliations:** 1 Johns Hopkins University School of Medicine, Baltimore, MD, USA; 2 Yale School of Medicine, Yale Center for Clinical Investigation, New Haven, CT, USA; 3 The Multi-Regional Clinical Trials Center of Brigham and Women’s Hospital and Harvard, Brigham and Women’s Hospital, Boston, USA

**Keywords:** Clinical trials, health policy, trial registration, results reporting, academic medical Center

## Abstract

**Introduction:**

Historically US-based academic organizations dedicated limited resources, including policies, personnel to ensuring compliance with clinical trials registration and results reporting requirements. A recent follow-up survey finds that 6-years after an initial survey, there is increased attention and dedication of resources to improve compliance rates for clinical trials registration and results reporting.

**Methods:**

Internet-based online survey using Qualtrics between 20 April 2023 and 30 September 2023 distributed to Protocol Registration and Results Reporting (PRS) Administrators at US-based academic organizations with ClinicalTrials.gov organizational accounts. The survey focused on the 249 respondents of the original 2016–2017 survey published in 2018. The overall response rate was 162/249 (65.06%) with 100% participation from National Cancer Center (NCI) Designated Cancer Centers and hubs of the Clinical and Translational Science Awards (CTSA).

**Results:**

Results indicated a marked increase of academic organizations with policies in place for registration (43 to 74%) and results reporting (35 to 68%). The median number of Full-time Equivalent (FTE) staff at responding academic organizations increased (from 0.08 to 0.5) with statistically significant difference between the number of organizational records and FTEs supporting registration and results reporting. Larger gains are seen with NCI-Designated Cancer Centers and/or CTSA hubs.

**Conclusions:**

It appears academic organizations are more equipped to comply with requirements, and demonstrate a trend towards appropriate staffing. In the 6 years since the original survey, US-based academic organizations have significantly increased attention to compliance with clinical trials registration and results reporting requirements, indicated by an increase in institutional policies and dedicated personnel.

## Introduction

Clinical trial transparency and disclosure is an ethical and scientific imperative, a tenant of building public trust and required for responsible stewardship of research funding. ClinicalTrials.gov is a public database run by the US National Library of Medicine (NLM) and provides detailed information about clinical studies. The database enables registration and reporting of individual trials, recruitment, data sharing statements and systematic reviews [1–3]. The US Food and Drug Administration Amendments Act of 2007 (FDAAA), also known as the Final Rule and National Institutes of Health (NIH) Policy on the Dissemination of NIH-Funded Clinical Trial Information have been in place since 2017 (Table 1).

**Table 1.** Historical policies that have impacted ClinicalTrials.gov

Year	Action
1997	The Food and Drug Administration Modernization Act of 1997 led to the creation of ClinicalTrials.gov
2000	National Library of Medicine (NLM) launches ClinicalTrials.gov
2004	International Committee of Medical Journal Editors (ICMJE) announced that trials initiated from 2005 would have to be registered to be considered for publication
2007	Title VIII of the Food and Drug Administration Amendments Act of 2007 (FDAAA) [31] required that certain trials of drugs, biologics, and medical devices be registered and that results for trials of approved products be posted on ClinicalTrials.gov Title VIII of the Food and Drug Administration Amendments Act of 2007 (FDAAA) [31] required that certain trials of drugs, biologics, and medical devices be registered and that results for trials of approved products be posted on ClinicalTrials.gov
2017	The Final Rule,,” which took effect on January 18, 2017, clarified and expanded requirements for registration and reporting
2017	The National Institutes of Health (NIH) issued broader requirements that apply to *all* trials funded by the NIH, including early trials and trials of behavioral interventions
2019	Effective January 21, 2019 the Common Rule revised Section 46.116 to require uploading the Informed Consent Form (ICF)

In 2018 members of the Clinical Trials Registration and Results Reporting Taskforce (Taskforce) [4] published a manuscript on the policies, procedures, and resources to support trial registration and reporting at Academic Medical Centers (AMCs) [5]. The results showed varied preparation for Final Rule implementation. For example, only 43% of academic organizations had a registration policy and 35% had a results reporting policy. The prior study intentionally sought the broadest possible respondent base and was successful in reaching accounts that comprised 85% of all University/Organization records at the time (40,351 of 47,701 records). The approach may have yielded an over representation of smaller accounts (<20 records posted on ClinicalTrials.gov), many of which did not have established policies and low Full-time Equivalent staff (FTE) support. The median FTE from this study has been cited by others as evidence that academic organizations are not doing enough to ensure compliance with federal requirements [6,7].

Several articles calling attention to noncompliance have been published and many organizations are raising the alarm on behalf of patients, research participants, science, and stewardship [8,9,10]. STAT News was among the first to broadcast the exact names and compliance metrics at 40 institutions [11]. A follow-up report from 2020 [12] revealed large gains at those same institutions, as was suggested in a 2019 commentary, “external pressure might incentivize improved reporting” [13].

Since 2018, research has shown improvements to the lagging compliance rates [14]. The Medical University of South Carolina reported a 98% increase in compliance after 12 months following the implementation of various strategies, including hiring a full-time ClinicalTrials.gov Administrator to assist researchers and implementing Institutional Review Board (IRB) workflows [15]. Johns Hopkins University also reported significant improvements in compliance after hiring 3 dedicated staff and formalizing a policy for registration and results reporting [16]. Despite growing efforts, academic organizations still perform below Industry in registering trials prospectively [17,18] and reporting results, however outpace the results compliance of government agencies [8,10].

## Federal initiatives

In August 2022 an Office of Inspector General (OIG) audit concluded that “NIH did not ensure that all NIH-funded Intramural and Extramural clinical trials complied with Federal reporting requirements” [19]. Since the audit, NIH has confirmed their commitment to increased communication and, “if needed, enforcement actions” [20]. The NIH Office of Policy for Extramural Research Administration (OPERA) has issued over 300 letters of potential noncompliance to grantee organizations. FDA has created a website to post Notices of Noncompliance as well as Pre-notice letters for voluntary corrective action, including unredacted copies of each letter [21]. At the time of publication of this manuscript, the FDA civil monetary penalties, adjusted for [22] annual inflation are $14,724 per study per day [23].

Lastly, NLM has been actively engaged in a multi-year, comprehensive effort to modernize the public ClinicalTrials.gov website and the Protocol Registration and Results Reporting System (PRS) [24]. The modernization of the PRS has provided interested parties an opportunity to collaborate with NLM to provide constructive feedback and rework internal guidance documents to enable re-learning for all Investigators and study teams to effectively navigate the new platforms.

The goal of the current survey was to understand if federal initiatives, public attention, and time has led to improvements in academia’s performance in meeting the requirements of clinical trials registration and results reporting. Researchers sought to quantify the changes in institutional support since the initial survey with respect to ClinicalTrials.gov policies and identify drivers for compliance improvements. Additionally, we explored if those academic organizations that are National Cancer Center (NCI) Designated Cancer Centers and/or hubs of the Clinical and Translational Science Awards (CTSA) [25,26], by virtue of federal funding and focused collaboration, have performed better than other institutions. CTSA hubs receive research infrastructure funding to, “develop, demonstrate and disseminate scientific and operational innovations” and may have additional ability to provide more resources, and potentially be under more scrutiny to have robust, local ClinicalTrials.gov programs and contribute to national dissemination efforts [27].

## Materials and methods

The 2023 survey was sent utilizing Qualtrics software (https://www.qualtrics.com/) and was adapted from the initial 2016–2017 survey. The questionnaire focused on local institutional polices, staffing resources, available tools, determining compliance and oversight of clinical trials registration and results reporting requirements. The 2023 survey was updated to include new questions since the last survey (e.g. Informed Consent Form requirements) and to better capture FTE resources at the institution (A pdf of the complete survey is posted as a supplemental document)

The NLM maintains ClinicalTrials.gov as two separate databases. One is for the general public to access published records. The PRS allows one or more PRS Administrators (Admins) to enter records for NLM review. Admins oversee registration and results reporting at academic sites. A health system may have only one University/Organization PRS Account, or several in cases where policy, procedures and/or staffing are distinct.

Recipients were identified using the list of respondent emails from the original survey. Given the several years in between administration of the surveys, many emails did not reach their destination. The authors utilized the professional network of the Taskforce as well as contacts at the local CTSA to identify a PRS Administrator at all previous respondent PRS accounts. Participants were emailed a new invitation to complete the survey and were able to request a completed version of their responses be returned to them as a pdf.

## Analytical approach

All responses were summarized overall using descriptive statistics, frequency and percentage for categorical responses and mean and standard deviation (or median and range) for continuous data.

Subgroup comparisons were performed based on PRS account size (number of study records). Between group comparisons were performed for institutions with and without CTSA Program funding and institutions with and without NCI-designated cancer centers.

Between subgroup comparisons of continuous variables were performed using either student *t* test or Mann–Whitney *U* test for 2 groups; ANOVA or Kruskal Wallace when 2 or more subgroups. Comparisons of categorical outcomes between subgroups were performed with chi-square or Fischer Exact tests. A p value of 0.05 was used to establish statistical significance. All data were analyzed using SPSS version 29.02 (IBM Corp. (2023). IBM SPSS Statistics for Windows, Version 29.02 [Computer software]. IBM Corp.).

## Results

The survey was open between 20 April 2023 and 30 September 2023. Two-hundred and forty-nine survey invitations were sent via email to PRS administrators at US-based academic organizations. A total of 162 (65.06%) survey responses were received. Within that overall number, we received a 100% response rate from NCI-Designated Cancer Centers (73/73) (75 total PRS accounts) and CTSA hubs (65/65) (85 total PRS accounts).

The following summarizes responses on institutional policies for clinical trials registration, result reporting, staffing resources, institutional tools, and compliance efforts.

## Institutional policies

Seventy-four percent of academic organizations confirmed having a registration policy, while 19.1% did not, and 4.3% were unsure. A small portion, 2.5%, did not answer the question. Concerning the timing of trial registrations, 30.0% required registration before enrollment begins, 27.5% within 21 days of beginning enrollment and 13.3% before IRB approval. Additionally, 20.8% had varying requirements based on trial type, 5.0% mentioned it was not addressed in the policy, less than 1% did not know or did not provide an answer to the question.

When asked to choose from a list of entities who held responsibility for determining whether a trial must be registered, the top responses were the PI (56.7%), PRS administrator (53.3%), and the IRB (32.5%). Some policies designated “other” responsible parties (11.7%), or did not assign this responsibility (2.5%), with 1.7% of respondents not knowing and 0.8% not answering the question.

Regarding results reporting policies, 67.9% of organizations reported having a policy, 24.7% did not, 4.9% did not know, and 2.5% did not answer the question. Of those with a results reporting policy, 87.3% assigned monitoring compliance with legal requirements to the PRS administrator, 46.4% to the PI, and 4.5% to the IRB. “Other” unspecified parties were assigned responsibility in 8.1% of cases, with 1.8% reporting no assignment in the policy, 0.9% did not know, and 1.8% did not respond.

Thirty-four percent of respondents had institutional policies that addressed investigators leaving the organization, 49.4% did not and 12.3% did not know, and 4.3% did not respond to the question. Finally, regarding penalties for failing to register or report trial results, 55.0% of responding organizations could penalize investigators, 30.8% could not, and 14.2% did not know, with no respondents skipping this question.

## Institutional ClinicalTrials.gov staffing characteristics

The average FTE support reported was 0.63 (± 0.69 SD) and the median was 0.50, with an interquartile range of 0.10 to 1.00. When the reported FTE was stratified by the number of study records at an institution, staff allocation increased with the number of records. For organizations with fewer than 100 records, the mean was 0.09 (± 0.19 SD), median = 0.05 (range = 0.00 to 1.00); 100 to 500 records mean FTE was 0.61 (± 0.47 SD), median = 0.50 (range = 0.03 to 2.00); organizations with 501 to 1000 records reported a mean FTE of 0.80 (± 0.51 SD), median = 0.68 (range = 0.10 to 2.00); and finally those with >1000 records reported a mean FTE of 1.34 (± 0.98),median = 1.00 (range = 0.06 to 3.88).

**Figure 1.**
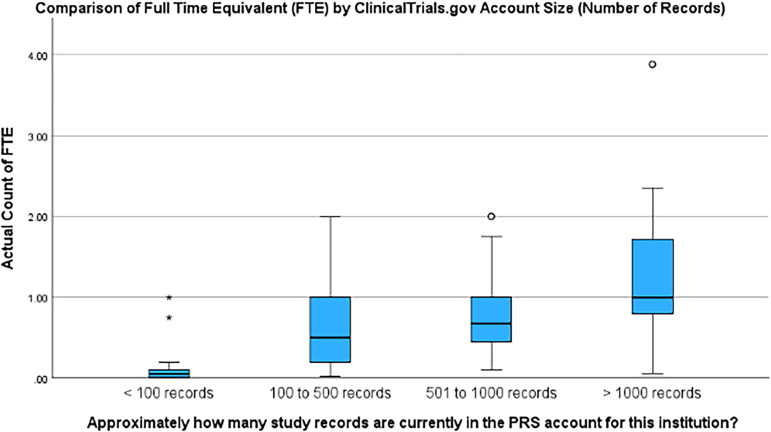
Comparison of full-time equivalent (FTE) by ClinicalTrials.gov account size (Number of records). Interpretation: the bold line in the middle of each of these boxplots represents the median value for each group. The symbols outside the upper portions of the boxplots represent outliers. The interior edges of the boxes represent the 25th and 75th quartiles. As the Kruskal–Wallis H tests were statistically significant, these figures are a representation of each of the four groups’ distributions of values across the 4 measures of FTE.

Of those studies that provided data on both the FTE staff at their institutions and their PRS record count (*n* = 150), a statistically significant difference was found with respect to the number of records and median FTE (*p < 0.001*). The difference between groups demonstrates a trend that as account sizes increase in record number, the number of staff at institutions also increases (Figure 1).

Staff supporting registration and results reporting efforts perform multiple functions, including: creating and maintaining user accounts (92.0%), reviewing problem records (90.7%), communicating with ClinicalTrials.gov staff (87.7%), notifying researchers about noncompliance sanctions (81.5%), enforcing compliance requirements (79.6%), transferring records between PRS accounts (77.8%), providing individualized support to investigators (74.7%), developing policies (74.1%), coordinating with internal groups (63.0%), and group training (53.7%), maintaining educational websites (46.9%), reviewing data management plans (35.8%) and NIH data dissemination plans (22.2%).

Additionally, dedicated staff performed specific functions related to registration including: responding to ClinicalTrials.gov compliance questions (90.1%), assisting with PRS review comments (88.3%), determining study registration requirements (77.2%), approving PRS account entries (70.4%), monitoring IRB-approved trial registrations (59.9%) and registering studies on behalf of investigators (43.8%).

Similarly, dedicated staff performed functions specific to results reporting including: responding to ClinicalTrials.gov compliance questions (88.9%), assisting with PRS review comments (86.4%), determining which studies require result reporting (72.8%), monitoring reporting requirements for ongoing IRB-approved trials (71.0%), approving PRS account entries (63.0%), and entering results on behalf of PIs (46.3%).

Staff qualifications among support staff were: high school (1.5%), associate’s degree (6.2%), bachelor’s degree (43.8%), master’s degree (43.1%), and higher degrees (34.6%).

## Tools and compliance

More than half of the organizations (52.5%) have an electronic system for managing ClinicalTrials.gov compliance, whereas 43.2% do not, with 1.9% unsure and 2.5% did not respond. Looking at the size of the PRS accounts (number of records), those with fewer than 100 records had the lowest adoption rates for electronic systems at 27.9%, while organizations with more than 1000 records had the highest at 81.5%.

Nineteen percent of respondents were not aware if their institution had a policy for the 2018 Common Rule requirement to upload the Informed Consent Form (ICF).

The survey also revealed that compliance tools developed since the initial survey are being adopted. A checklist tool is now being used at 56/162, (34.6%) of respondent institutions [28].

### CTSA analyses

Among CTSAs, 80.0% had a registration policy, compared to 70.2% of non-CTSAs. Regarding results reporting policies, 78.4% of CTSAs had one, versus 61.4% of non-CTSAs. Additionally, 65.9% of CTSAs had an electronic system for managing trial registration or results reporting, while only 35.1% of non-CTSAs did. The difference in FTE staff dedicated to these tasks at CTSAs was significantly higher (0.60 compared to 0.23, *p* = 0.003) (Table 2).

**Table 2.** Comparison of full-time equivalent staff by subgroup

Sub-Group	Mean (± SD)	Median (Min, Max)	*p* value
Did the institution have funding from the Clinical Translational Science Award (CTSA)?			
Yes (*N* = 85)	0.75 (± 0.77)	0.60 (0.00, 3.88)	0.003*
No (*N* = 68)	0.47 (± 0.52)	0.23 (0.00, 2.00)	
Does your institution have a Cancer Center affiliated with the National Cancer Institute?			
Yes (*N* = 73)	0.93 (± 0.78)	0.80 (0.02, 3.88)	<0.001*
No (*N* = 80)	0.35 (± 0.42)	0.12 (0.00, 2.00)	

*p value based on results of Mann–Whitney *U* test testing the median values.

Sub-Group analysis of institutions that are participating hubs of the Clinical Translational Science Award (CTSA) and are NCI-Designated Cancer Centers. Both showed statistically significant differences in the number of Full-Time Equivalent Staff (FTE).

Abbreviations: CTSA = Clinical Translational Science Award; NCI = National Cancer Institute; FTE = Full-Time Equivalent.

### NCI analyses

Comparing NCI cancer centers to non-cancer centers among the organizations surveyed, 88.0% of cancer centers had a registration policy versus 65.1% of non-cancer centers. In terms of a results reporting policy, 81.3% of cancer centers had one, compared to 59.0% of non-cancer centers. A majority of cancer centers (66.7%) also had an electronic system, 42.2% of non-cancer centers did not have one. The median FTE staff for these tasks was higher in cancer centers at 0.80 FTE compared to 0.12 FTE in non-cancer centers. Overall, NCI respondents reported a statistically significant higher median of FTE dedicated to ClinicalTrials.gov (*p* < 0.001) (Table 2).

## Discussion

Utilizing a similar survey as the one published in 2018, with the same academic organizations represented allows for a direct comparison of key policy elements over time (Table 3).

**Table 3.** Changes in ClinicalTrials.gov policies at university/Organization PRS accounts at the time of the initial survey and today

Institutional response	2018 Survey (*n* = 366)	2023 Survey (*n* = 165)
***For clinical trials related to the PRS account, does your organization have a written policy, standard operating procedure, or other form of written guidance regarding:***		
Registration policy *n*, %	156, 43%	122, 74%
Results reporting Policy *n*, %	129, 35%	112, 68%
PIs leaving the institution *n*, %	38, 24% *	58, 35%
ICF uploading *n*, %	N/A	55, 33%
***Full-Time equivalent (FTE) support for institutions***		
FTE support (overall number) median, (IQR)	0.08 (0.02–0.25)	0.50 (0.01–1.0)
FTE support (< 100 records) *n*; mean (SD)	199; 0.14 (0.37)	40; 0.09 (0.19)
FTE support (100–500 records) *n*; mean (SD)	70: 0.47 (0.45)	54; 0.61 (0.47)
FTE support (501–1,000 records) *n*; mean (SD)	13: 1.41 (1.48)	30; 0.80 (0.51)
FTE support (> 1,000 records) *n*; mean (SD)	5: 2.02 (0.56)	26; 1.34 (0.98)

Abbreviations: PRS = Protocol Registration and Results Reporting System; PI = Principal Investigator; ICF = Informed Consent Form; FTE = Full-Time Equivalent.

*(Denominator: *n* = 156).

These survey results provide evidence that since 2018, academic organizations have dedicated greater resources towards compliance with ClinicalTrials.gov requirements. In 2018, 43% of respondents identified an institutional policy regarding trial registration compared to 74% in 2023. Similar results for the NCI (88%) and/or CTSA (80%) institutions indicate that these types of institutions may place a greater emphasis on policy implementation and education [28]. With respect to policies related to results reporting, 35% of 2018 respondents identified having such a policy compared to 68% in 2023 (78.4 CTSAs, 81.3 NCI cancer centers respectively). Since the initial survey, institutions are more aware of the importance of clinical trial transparency and of the public pressure to accurately register and report results of clinical trials in a timely fashion. They have joined in community to develop and share more comprehensive policies (e.g., AMCs, with policies for PI’s leaving increased from 24 to 35%) and tools (e.g., Yale Clinical trial Dashboard [29]).

Further, since 2018, academic organizations report an overall increase in the FTE support dedicated to ClinicalTrials.gov activities (8% of an FTE in 2018, to 50% of an FTE in 2023). These increases appear to be consistent across the size (# of records) of the academic organizations’ PRS account. Our findings indicate a statistically significant difference between account size groupings (*p < 0.001*). These data indicate that not only are institutions providing more support in terms of policies for staff and faculty to follow, but also with the appropriate staffing to support ClinicalTrials.gov.

Questions with a high percentage of respondents being unaware of the answer indicate opportunities for best practice dissemination or a need for institutional reinforcement of these policies.

## Drivers of change

The results of this survey could also lead to targeted educational outreach to inform and provide policy language, leading to standardized processes and improved compliance to federal requirements. Investigators and study teams understand the need for transparency and want to report their research but may lack the knowledge and resources needed to fully comply.

Having a senior level Project Champion at each AMC establishing and enforcing strong policies could also be a major driver of positive change. There is also a role for strategic funding and directives of CTSA leadership. We know that formal communication from FDA is effective [21]. The effectiveness of civil monetary penalties is unknown. Until the first penalty is issued, PRS Administrators use any updates of policy, the pre-notice website and every annual inflation adjustment [30] to keep everyone aware of financial, civil and criminal penalties.

## Limitations

The original survey was facilitated by an institutional contacts list provided by NLM for all University/Organization PRS Accounts. The authors were not able to obtain an institutional contacts list in 2023. Efforts were therefore undertaken to manually acquire updated contact information for prior respondents, leading to a significant delay in resending the survey.

## Conclusions

Academic organizations must renew their efforts to implement strong policies and work collaboratively with colleagues across academia. Transparency advocates continue to highlight the importance of clinical trial reporting and the impact of holding academia accountable. Collectively we ensure, not just compliance with all the regulations, but to honor the spirit of transparency behind them. Increased institutional FTE support will be needed to implement best practices, policies and procedures and to educate investigators and study teams in navigating the modernized ClinicalTrials.gov platform. The more active engagement from PRS Administrators at academic sites the better the modernized ClinicalTrials.gov platform will function for all. The authors want to acknowledge the importance of advocacy work here in the United States [31,32] and around the world (e.g., The FDAAA Trials Tracker [33], the United Kingdom’s, International Clinical Trials Registry Platform [34]). Collectively, these efforts have been successful in highlighting the importance of transparency, scientific vigor and stewardship. We consider these groups as partners and have welcomed them to share their efforts with the Taskforce.

### Taskforce

The Clinical Trials Registration and Results Reporting Taskforce is a network of academic organizations whose representatives meet monthly by teleconference, share resources, and provide informal peer education. Since 2014, the Taskforce has grown to over 700 members at over 250 US-based academic organizations and includes governmental agencies and other relevant partners. Nearly 200 members engage in monthly webinars. The Taskforce continues to advance best practices and develop and disseminate key deliverables as well as serves as an international collaborator for global clinical trial disclosure initiatives [35].

## Supporting information

10.1017/cts.2025.10171.sm001 Keyes et al. supplementary material Keyes et al. supplementary material

## Data Availability

The statistical code for generating these results is available from the authors. We did not obtain consent to identify participants.

## Supplementary material

The supplementary material for this article can be found at https://doi.org/10.1017/cts.2025.10171.

## References

[1] Jiang L , Vorland C , Ying X , et al. SPIRIT-CONSORT-TM: a corpus for assessing transparency of clinical trial protocol and results publications. Sci Data. 2025;12:04629. doi: 10.1038/s41597-025-04629-1. PMC11871027 40021657

[2] TranspariMED. NIH research waste: billions lost due to unreported clinical trial results. TranspariMED Website Published 2025 (https://www.transparimed.org/single-post/nih-research-waste) Accessed March 27, 2025.

[3] Statham EE , White SA , Sonwane B , Bierer BE. Primed to comply: individual participant data sharing statements on ClinicalTrials.gov. PLoS One. 2020;15:e0226143. doi: 10.1371/journal.pone.0226143. 32069305 PMC7028256

[4] Clinical Trials Registration and Results Reporting Taskforce (CTRR Taskforce). Official website., CTRR Taskforce. (https://ctrrtaskforce.org/) Accessed March 27, 2025.

[5] Mayo-Wilson E , Heyward J , Keyes A , et al. Clinical trial registration and reporting: a survey of academic organizations in the United States. BMC Med. 2018;16:60. doi: 10.1186/s12916-018-1042-6. 29716585 PMC5930804

[6] Zarin DA , Fain KM , Dobbins HD , Tse T , Williams RJ. 10-year update on study results submitted to ClinicalTrials.gov. N Engl J Med. 2019;381:1966–1974. doi: 10.1056/NEJMsr190764457. 31722160 PMC8591666

[7] Newswise. Most academic institutions unprepared to meet new HHS clinical trial reporting regs. Newswise Website Published April 30. 2018. (https://d.newswise.com/articles/view/693709/?sc=rsla) Accessed March 27, 2025.

[8] Piller C. FDA and NIH let clinical trial sponsors keep results secret and break the law. *Science Published January 13*, 2020. (https://www.science.org/content/article/fda-and-nih-let-clinical-trial-sponsors-keep-results-secret-and-break-law) Accessed March 27, 2025.

[9] Silverman E. Advocacy groups petition FDA for greater transparency in clinical trial reporting. *STAT Published February*. 2023. (https://www.statnews.com/pharmalot/2023/02/27/fda-petition-clinical-trials-transparency-nih/) Accessed March 27, 2025.

[10] DeVito NJ , Bacon S , Goldacre B. Compliance with legal requirement to report clinical trial results on ClinicalTrials.gov: a cohort study. Lancet. 2020;395:361–369. doi: 10.1016/S0140-6736(19)33220-9. 31958402

[11] Kaplan S. NIH promises to crack down on clinical trial reporting violations. STAT. Published January 9, 2018. (https://www.statnews.com/2018/01/09/clinical-trials-reporting-nih/) Accessed March 27, 2025.

[12] TranspariMED. New report: 25 major U.S. medical universities violate key transparency law. TranspariMED website. Published March 25, 2019. (https://www.transparimed.org/single-post/2019/03/25/New-report-25-leading-US-universities-violate-key-medical-transparency-law) Accessed March 27, 2025.

[13] Zarin DA. The culture of trial results reporting at academic medical centers. JAMA Intern Med Published October. 2029;180:319. doi: 10.1001/jamainternmed.2019.4200. 31657846

[14] Universities Allied for Essential Medicines (UAEM). Clinical Trials Transparency Report. Altreroute website. Published 2020. (https://altreroute.com/clinicaltrials/assets/download/Clinical_Trials_Transparency_Report_UAEM_v5.pdf) Accessed March 27, 2025.

[15] Snider SH , Flume PA , Gentilin SL , Lesch WA , Sampson RR , Sonne SC. Overcoming non-compliance with clinical trial registration and results reporting: one institution’s approach. Contemp Clin Trials Commun. 2020;18:100557. doi: 10.1016/j.conctc.2020.100557. 32258818 PMC7118290

[16] Keyes A , Mayo-Wilson E , DPhil PN , Lalji A , Tetteh O , Ford DE. Creating a program to support registering and reporting clinical trials at Johns Hopkins University. Acad Med. 2021;96:527–533. doi: 10.1097/ACM.0000000000003806. PMC8012215 33060401

[17] Zarin DA , Tse T , Williams RJ , Rajakannan T. Update on trial registration 11 years after the ICMJE policy was established. N Engl J Med. 2017;376:383–391. doi: 10.1056/NEJMsr1601330. 28121511 PMC5813248

[18] Law MR , Kawasumi Y , Morgan SG. Despite law, fewer than one in eight completed studies of drugs and biologics are reported on time on ClinicalTrials.gov. Health Aff (Millwood). 2011;30:2338–2345. doi: 10.1377/hlthaff.2011.0061. 22147862

[19] US Department of Health and Human Services Office of Inspector General. The National Institutes of Health did not ensure that all clinical trial results were reported in accordance with federal requirements. Published August 2022. (https://oig.hhs.gov/oas/reports/region6/62107000.asp) Accessed March 27, 2025.

[20] Lauer M. NIH clinical trials reporting compliance: a shared commitment. NIH Office of the Director. Published March 24 2023, (https://nexus.od.nih.gov/all/2023/03/24/nih-clinical-trials-reporting-compliance-a-shared-commitment/) Accessed March 27, 2025.

[21] Ramachandran R , Morten CJ , Ross JS. Strengthening the FDA’s enforcement of ClinicalTrials.gov reporting requirements. JAMA. 2021;326:2131–2132. doi: 10.1001/jama.2021.19773. 34766971

[22] US Food and Drug Administration. Civil money penalties relating to the ClinicalTrials.gov data bank: Guidance for industry. 2025. (https://www.fda.gov/regulatory-information/search-fda-guidance-documents/civil-money-penalties-relating-clinicaltrialsgov-data-bank) Accessed March 27, 2025.

[23] Electronic Code of Federal Regulation. Definitions, 45 CFR § 102.3. Published 2025. (https://www.ecfr.gov/current/title-45/subtitle-A/subchapter-A/part-102/section-102.3) Accessed March 27, 2025.

[24] ClinicalTrials.gov. ClinicalTrials.gov modernization. ClinicalTrials.gov website. Published March 2025. (https://clinicaltrials.gov/about-site/modernization) Accessed March 27, 2025.

[25] Clinical and Translational Science Awards (CTSA) Program. ACTSA Program hub directory. CTSA website. (https://ccos-cc.ctsa.io/resources/hub-directory) Accessed March 27, 2025.

[26] Published. National cancer institute. NCI-designated cancer centers. National Cancer Institute website. Published March 19, 2025, Accessed March 27, 2025.

[27] O’Reilly EK , Hassell NJ , Snyder DC , et al. ClinicalTrials.gov reporting: strategies for success at an academic health center. Clin Transl Sci. 2015;8:48–51. doi: 10.1111/cts.12235. 25387802 PMC4329023

[28] Tetteh O , Nuamah P , Keyes A. Addressing the quality of submissions to ClinicalTrials.gov for registration and results posting: the use of a checklist. Clin Trials Published August. 2020;17:717–722. doi: 10.1177/1740774520942746. PMC7655525 32755266

[29] Maciejewski K. ClinicalTrials.gov Dashboard. ShinyApps.io. (https://kmaciejewski.shinyapps.io/CTGovDashboard/) Accessed April 10, 2025.

[30] US Department of Health and Human Services. Annual civil monetary penalties inflation adjustment. Fed Regist. Published August 8, 2024. (https://www.federalregister.gov/documents/2024/08/08/2024-17466/annual-civil-monetary-penalties-inflation-adjustment) Accessed April 30, 2025.

[31] TranspariMED. FDAAA enforcement and representative Pallone’s call for stronger action.TranspariMED website. Published 2022. (https://www.transparimed.org/single-post/fdaaa-pallone) Accessed March 27, 2025.

[32] Universities Allied for Essential Medicines (UAEM). Clinical trials transparency report, Altreroute website. Updated 2024. (https://altreroute.com/clinicaltrials/assets/download/Clinical_Trials_Transparency_Report_UAEM_v5.pdf) Accessed March 27, 2025.

[33] University of Oxford. Evidence-Based Medicine DataLab. FDAAA trialsTracker. (http://fdaaa.trialstracker.net/) Accessed March 27, 2025.

[34] Silverman E. U.K. Becomes the first country to release detailed data on sponsors that fail to register clinical trials. STAT News. Published March 28, 2024. (https://www.statnews.com/pharmalot/2024/03/28/uk-studies-research-transparency-trials) Accessed March 31, 2025.

[35] Franzen DL , Salholz-Hillel M , Müller-Ohlraun S , Strech D. Improving research transparency with individualized report cards: a feasibility study in clinical trials at a large university medical center. BMC Med Res Methodol. 2025;25:2482. doi: 10.1186/s12874-025-02482-9. PMC11823227 39948475

