# Supplementary material for: Evaluating the current landscape of clinical trials registration and results reporting policies, procedures and staffing at US-based academic centers: Survey revisited
Source: J Clin Transl Sci. 2025 Oct 13;9(1):e251. doi: 10.1017/cts.2025.10171 (PMC12695459; doi:10.1017/cts.2025.10171)
Supplement: Keyes et al. supplementary material [file S2059866125101714sup001.pdf]

Section 1: Consent

Study Title: Survey for Protocol Registration and Results System (PRS) Administrators

Research Study Summary:

The purpose of this research study is to gain further understanding about ClinicalTrials.gov registration and reporting policies and practices. We have invited PRS administrators at academic medical centers, universities, and other similar research organizations in the United States to participate. It has been discussed and promoted on monthly meetings of the Clinical Trials Registration and Results Reporting Taskforce (CTRRT).

Consent:

Study activities will include: answering questions regarding ClinicalTrials.gov registration and reporting policies and practices via electronic survey.

Your involvement will require: approximately 15 minutes to complete this survey. If you cannot complete all the questions now, you may return to complete it later.

We do not expect any risks from taking part in this study. If you wish to keep your responses anonymous and/or any sharing of identifying information regarding the institution that you are employed by, you may do so.

The study may have no benefits to you, however, your participation in this survey will be used to inform the larger conversation regarding ClinicalTrials.gov compliance at academic medical centers, universities, and other similar research organizations in the United States.

Taking part in this study is your choice. You can choose to take part, or you can choose not to take part in this study. You also can change your mind at any time. Whatever choice you make will not have any effect on your relationship with the CTRRT.

If you are interested in learning more about the study, please continue reading, or have someone read to you, the rest of this document. Ask the study staff questions about anything you do not understand.

Are there any costs to participation? Will I be paid for participation?
You will not have to pay for taking part in this study. You will not be paid for taking part in this study.

How will you keep my data safe and private?
All of your responses will be held in confidence or anonymous if you elect. Only the researchers involved in this study and those responsible for research oversight (such as representatives of the Yale University Human Research Protection Program, the Yale University Institutional Review Boards, and others) will have access to any information that could identify you that you provide. We will share it with others if you agree to it or when we have to do it because U.S. or State law requires it. For example, we will tell somebody if we learn that you are hurting a child or an older person.

Data will be collected using a secure platform and survey data will be downloaded and stored on a secure server. ID numbers will be assigned to responses and any identifying information linking responses to individuals will be kept in a separate file matching the ID numbers to the identity of the

respondent and institution. This information will only be collected for the purposes of monitoring survey response rate and following up with those that have not completed the survey fully or have not completed the survey at all. When we publish the results of the research or talk about it in conferences, we will not use your name. We will also share information about you with other researchers for future studies, but we will not use your name or any other identifiers. We will not ask you for any additional permissions.

What if I want to refuse or end participation before the study is over?
Taking part in this study is your choice. You can choose to take part, or you can choose not to take part in this study. You also can change your mind at any time. Whatever choice you make will not have any effect on your relationship with CTRRT. You do not give up any of your legal rights by giving your verbal agreement to participate.

Who should I contact if I have questions?
Please feel free to ask about anything you don't understand. If you have questions later or if you have a research-related problem, you can call the Principal Investigator at 203-824-2356.

If you have questions about your rights as a research participant, or you have complaints about this research, you call the Yale Institutional Review Boards at (203) 785-4688 or email hrpp@yale.edu.

Section 2: Eligibility

1a. Is your organization located in the United States of America?

- ○ Yes
- ○ No

1b. Are trials in the PRS account "${m://ExternalDataReference}" conducted in the United States of America?

- ○ Yes
- ○ No

2a. What types of organizations use the PRS account "${m://ExternalDataReference}"? Check all that apply.

- ☐ Cancer center: NCI designated
- ☐ Cancer center: Not NCI designated
- ☐ Cardiovascular institute
- ☐ Community mental health center
- ☐ Contract research organization (CRO)
- ☐ Hospital(s) or hospital network(s): For-profit
- ☐ Hospital(s) or hospital network(s): Non-profit
- ☐ Individual investigator
- ☐ Manufacturer (e.g. drug, device)
- ☐ Private medical practice
- ☐ Research organization: For-profit
- ☐ Research organization: Non-profit
- ☐ University, college, or school: All parts of the organization
- ☐ University, college, or school: School(s) or department(s) within the organization
- ☐ Other
- ☐ Don't know

2b. What other types of organization(s) uses the PRS account

"${m://ExternalDataReference}"?

2c. Which school(s) or department(s) use the PRS account "${m://ExternalDataReference}"? Check all that apply.

- ☐ Arts and sciences
- ☐ Chiropractic
- ☐ Dentistry
- ☐ Education
- ☐ Engineering
- ☐ Family and consumer sciences
- ☐ Graduate studies
- ☐ Health professionals
- ☐ Health sciences
- ☐ Medicine
- ☐ Nursing
- ☐ Optometry
- ☐ Pharmacy
- ☐ Physical therapy
- ☐ Physicians and surgeons
- ☐ Psychology
- ☐ Public health
- ☐ Public policy
- ☐ Social work

- [ ] Veterinary medicine
- [ ] Other

2d. What other school(s) or department(s) uses the PRS account "${m://ExternalDataReference}"?

4a. You answered "Don't know" to one of the previous questions. Would you prefer that we contact someone else at your organization to answer this survey?

- ○ Yes, I will suggest one of my colleagues.
- ○ No, I will complete the survey for my organization.

4b. Please enter the name and e-mail address of the person we should contact to answer this survey.

Section 3: PRS account characteristics

5a. Select the number of NCI designated cancer centers that use the PRS account "${m://ExternalDataReference}".

- 1
- 2
- 3
- 4
- 5
- 6
- 7
- 8
- 9
- 10
- More than 10

5b. Select the number of non-profit hospitals or hospital networks that use the PRS account "${m://ExternalDataReference}".

- 1
- 2
- 3
- 4
- 5
- 6
- 7
- 8
- 9
- 10
- More than 10

5c. Select the number of for-profit hospitals or hospital networks that use the PRS account "${m://ExternalDataReference}".

- 1
- 2
- 3
- 4
- 5
- 6
- 7
- 8
- 9
- 10
- More than 10

5d. Select the number of schools of medicine that use the PRS account "${m://ExternalDataReference}".

- 1
- 2
- 3
- 4
- 5
- 6
- 7
- 8
- 9
- 10

○ More than 10

5e. Select the NCI designated cancer center that uses the PRS account "${m://ExternalDataReference}".

[▾]

5f. Select the NCI designated cancer centers that use the PRS account "${m://ExternalDataReference}". Check all that apply.

☐ Abramson Cancer Center
☐ Albert Einstein Cancer Center
☐ Alvin J. Siteman Cancer Center
☐ Arizona Cancer Center
☐ Barbara Ann Karmanos Cancer Institute
☐ Cancer Therapy & Research Center
☐ Case Comprehensive Cancer Center
☐ Chao Family Comprehensive Cancer Center
☐ City of Hope Comprehensive Cancer Center
☐ Cold Spring Harbor Laboratory Cancer Center
☐ Comprehensive Cancer Center James Cancer Hospital & Solove Research Institute
☐ Dan L Duncan Comprehensive Cancer Center
☐ Dana Farber / Harvard Cancer Center
☐ David H. Koch Institute for Integrative Cancer Research at MIT
☐ Duke Cancer Institute
☐ Fox Chase Cancer Center
☐ Fred & Pamela Buffett Cancer Center
☐ Fred Hutchinson / University of Washington Cancer Consortium
☐ Georgetown Lombardi Comprehensive Cancer Center

- [ ] Greenebaum Cancer Center
- [ ] Harold C. Simmons Comprehensive Cancer Center
- [ ] Herbert Irving Comprehensive Cancer Center
- [ ] Holden Comprehensive Cancer Center
- [ ] Hollings Cancer Center
- [ ] Huntsman Cancer Institute
- [ ] Indiana University Melvin & Bren Simon Cancer Center
- [ ] Jonsson Comprehensive Cancer Center
- [ ] Markey Cancer Center
- [ ] Masonic Cancer Center
- [ ] Massey Cancer Center
- [ ] Mayo Clinic Cancer Center
- [ ] MD Anderson Cancer Center
- [ ] Memorial Sloan Kettering Cancer Center
- [ ] Moffitt Cancer Center
- [ ] Moores Comprehensive Cancer Center
- [ ] Norris Cotton Cancer Center
- [ ] NYU Cancer Institute
- [ ] OHSU Knight Cancer Institute
- [ ] Purdue University Center for Cancer Research
- [ ] Robert H. Lurie Comprehensive Cancer Center
- [ ] Roswell Park Cancer Institute
- [ ] Rutgers Cancer Institute of New Jersey
- [ ] Salk Institute Cancer Center
- [ ] Sanford Burnham Prebys Medical Discovery Institute
- [ ] Sidney Kimmel Cancer Center at Thomas Jefferson University
- [ ] Sidney Kimmel Comprehensive Cancer Center

- [ ] St. Jude Children's Research Hospital
- [ ] Stanford Cancer Institute
- [ ] The Jackson Laboratory Cancer Center
- [ ] The Wistar Institute Cancer Center
- [ ] Tisch Cancer Institute
- [ ] UAB Comprehensive Cancer Center
- [ ] UC Davis Comprehensive Cancer Center
- [ ] UCSF Helen Diller Family Comprehensive Cancer Center
- [ ] UNC Lineberger Comprehensive Cancer Center
- [ ] University of Chicago Comprehensive Cancer Center
- [ ] University of Colorado Cancer Center
- [ ] University of Hawaii Cancer Center
- [ ] University of Kansas Cancer Center
- [ ] University of Michigan Comprehensive Cancer Center
- [ ] University of New Mexico Cancer Research & Treatment Center
- [ ] University of Pittsburgh Cancer Institute
- [ ] University of Wisconsin Carbone Cancer Center
- [ ] USC Norris Comprehensive Cancer Center
- [ ] UVA Cancer Center
- [ ] Vanderbilt Ingram Cancer Center
- [ ] Wake Forest Comprehensive Cancer Center
- [ ] Winship Cancer Institute of Emory University
- [ ] Yale Cancer Center
- [ ] Other

5g. What other cancer center(s) uses the PRS account "${m://ExternalDataReference}"?

5h. Select the hospital or hospital network (e.g. Mayo Health Network) that uses the PRS account "${m://ExternalDataReference}"

5i. Select the hospitals or hospital networks (e.g. Mayo Health System) that use the PRS account "${m://ExternalDataReference}". Check all that apply.

- ☐ Advocate Christ Medical Center
- ☐ Advocate Health Care
- ☐ Advocate Illinois Masonic Medical Center
- ☐ Advocate Lutheran General Hospital
- ☐ Akron Children's Hospital
- ☐ Akron General Medical Center
- ☐ Albany Medical Center Hospital
- ☐ Albany Veterans Affairs Medical Center
- ☐ Albert Einstein Medical Center (Albert Einstein Healthcare Network)
- ☐ All Children's Hospital
- ☐ Allegheny General Hospital
- ☐ Anne Arundel Medical Center
- ☐ Arkansas Children's Hospital
- ☐ Atlantic Health
- ☐ Augusta University Medical Center
- ☐ Augusta Veterans Affairs Medical Center

- ☐ Aurora Health Care
- ☐ Banner University Medical Center Phoenix
- ☐ Banner University Medical Center Tucson Campus
- ☐ Bannerhealth
- ☐ Baptist Health South Florida
- ☐ Barnabas Health
- ☐ Barnes Jewish Hospital
- ☐ Bassett Healthcare
- ☐ Baton Rouge General Medical Center
- ☐ Baylor Heart and Vascular Hospital
- ☐ Baylor St. Luke’s Medical Center
- ☐ Baylor University Medical Center
- ☐ Baystate Health System
- ☐ Baystate Medical Center
- ☐ Beaumont Dearborn
- ☐ Beaumont Health
- ☐ Beaumont Royal Oak
- ☐ Benioff Children's Hospital Oakland
- ☐ Berkshire Medical Center
- ☐ Beth Israel Deaconess Medical Center
- ☐ Bethesda North Hospital
- ☐ Birmingham Veterans Affairs Medical Center
- ☐ BJC HealthCare
- ☐ Blythedale Children's Hospital
- ☐ Boston Children's Hospital
- ☐ Boston Medical Center
- ☐ Bridgeport Hospital

- ☐ Brigham and Women's Hospital
- ☐ Cabell Huntington Hospital
- ☐ California Pacific Medical Center
- ☐ Cambridge Health Alliance
- ☐ Cambridge Health Alliance Cambridge Hospital Campus
- ☐ CAMC Health System
- ☐ Captain James A. Lovell Federal Health Care Center
- ☐ Care New England Health System
- ☐ CareGroup Inc.
- ☐ Carilion Medical Center
- ☐ Carolinas HealthCare System
- ☐ Carolinas Medical Center
- ☐ Cedars Sinai Medical Center
- ☐ Central Arkansas Veterans Healthcare System
- ☐ Central Texas Veterans Health Care Sys
- ☐ Charleston Area Medical Center
- ☐ CHI Health
- ☐ CHI Health Creighton University Medical Center Bergan Mercy
- ☐ CHI St. Luke’s Health
- ☐ Children's Healthcare of Atlanta
- ☐ Children's Healthcare of Atlanta (Includes Egleston and Scottish Rite)
- ☐ Children's Hospital Los Angeles
- ☐ Children's Hospital of Philadelphia
- ☐ Children's Hospital of Wisconsin
- ☐ Children's Medical Center of Dallas
- ☐ Children's Memorial Hospital
- ☐ Children's Mercy Hospital

- ☐ Children's National Medical Center
- ☐ Christiana Care Health System
- ☐ Cincinnati Children's Hospital Medical Center
- ☐ City of Hope National Medical Center
- ☐ Cleveland Clinic Foundation
- ☐ Cleveland Clinic Health System
- ☐ Community Health Network
- ☐ Community Regional Medical Center
- ☐ Cooper University Hospital
- ☐ Dana Farber Cancer Institute
- ☐ Danbury Hospital
- ☐ Dartmouth Hitchcock Alliance
- ☐ Dartmouth Hitchcock Medical Center
- ☐ Dayton Veterans Affairs Medical Center
- ☐ Denver Health and Hospital Authority
- ☐ Department of Veterans Affairs Veterans Integrated Service Network VISN 1
- ☐ Department of Veterans Affairs Veterans Integrated Service Network VISN 10
- ☐ Department of Veterans Affairs Veterans Integrated Service Network VISN 11
- ☐ Department of Veterans Affairs Veterans Integrated Service Network VISN 12
- ☐ Department of Veterans Affairs Veterans Integrated Service Network VISN 17
- ☐ Department of Veterans Affairs Veterans Integrated Service Network VISN 18
- ☐ Department of Veterans Affairs Veterans Integrated Service Network VISN 2
- ☐ Department of Veterans Affairs Veterans Integrated Service Network VISN 22
- ☐ Department of Veterans Affairs Veterans Integrated Service Network VISN 23
- ☐ Department of Veterans Affairs Veterans Integrated Service Network VISN 4
- ☐ Department of Veterans Affairs Veterans Integrated Service Network VISN 5
- ☐ Department of Veterans Affairs Veterans Integrated Service Network VISN 6

- ☐ Department of Veterans Affairs Veterans Integrated Service Network VISN 7
- ☐ Department of Veterans Affairs Veterans Integrated Service Network VISN 8
- ☐ Department of Veterans Affairs Veterans Integrated Service Network VISN 9
- ☐ Dignity Health
- ☐ Duke University Health System
- ☐ Duke University Hospital
- ☐ Durham Veterans Affairs Medical Center
- ☐ Edward W. Sparrow Hospital
- ☐ Emory Healthcare
- ☐ Emory University Hospital
- ☐ Emory University Hospital Midtown
- ☐ Englewood Hospital and Medical Center (EHMC)
- ☐ Erlanger Health System
- ☐ Erlanger Medical Center
- ☐ Eskenazi Health
- ☐ Fairview Health Services
- ☐ Florida Hospital Orlando
- ☐ Franklin Square Hospital
- ☐ Froedtert Hospital
- ☐ Geisinger Medical Center
- ☐ General Health System
- ☐ George Washington University Hospital
- ☐ Georgetown University Hospital
- ☐ Good Samaritan Hospital (TriHealth)
- ☐ Grady Memorial Hospital Atlanta GA
- ☐ Grandview Medical Center
- ☐ Grant Riverside Methodist Hospitals Grant Medical Center Campus

- [ ] Grant Riverside Methodist Hospitals Riverside Campus
- [ ] Greenville Health System
- [ ] Greenwich Hospital
- [ ] Gundersen Health System
- [ ] Gundersen Lutheran Medical Center
- [ ] Gwinnett Medical Center Lawrenceville
- [ ] Hackensack University Medical Center
- [ ] Harbor UCLA Medical Center
- [ ] Harborview Medical Center
- [ ] Hartford HealthCare
- [ ] HealthPartners Inc.
- [ ] Heart Hospital Baylor Plano
- [ ] Hennepin County Medical Center
- [ ] Henry Ford Health System
- [ ] Henry Ford Hospital
- [ ] HonorHealth Scottsdale Osborn Medical Center
- [ ] Hospital for Special Surgery
- [ ] Hospital of the University of Pennsylvania
- [ ] Houston Methodist
- [ ] Houston Methodist Hospital
- [ ] Howard University Hospital
- [ ] Hunter Holmes McGuire Veterans Affairs Medical Center
- [ ] Hurley Medical Center
- [ ] Indiana University Health Inc
- [ ] Indiana University Health Methodist Hospital
- [ ] INOVA Fairfax Hospital
- [ ] INOVA Health System

- ☐ Iowa City Veterans Affairs Medical Center
- ☐ Jackson Memorial Hospital
- ☐ James J. Peters VA Medical Center
- ☐ Jefferson Health System
- ☐ Jerry L. Pettis Memorial Veterans Affair Medical Center
- ☐ Jersey Shore University Medical Center
- ☐ Jesse Brown VA Medical Center
- ☐ John H. Stroger Jr. Hospital of Cook County
- ☐ John Peter Smith Hospital (Tarrant County Hospital District)
- ☐ Johns Hopkins Bayview Medical Center
- ☐ Johns Hopkins Health System
- ☐ Johns Hopkins Hospital
- ☐ Johnson City Medical Center
- ☐ Kaiser Foundation Hospital Los Angeles
- ☐ Kaiser Foundation Hospitals Northern California
- ☐ Kaiser Permanente Foundation Hospitals Southern California
- ☐ Keck Medical Center of USC
- ☐ KentuckyOne Health University of Louisville Hospital
- ☐ Kettering Medical Center
- ☐ Lahey Hospital and Medical Center
- ☐ Lancaster General Hospital
- ☐ Lankenau Hospital
- ☐ LeBonheur Children's Hospital
- ☐ Lehigh Valley Health Network
- ☐ Lehigh Valley Hospital Cedar Crest
- ☐ Lenox Hill Healthcare Network
- ☐ Lenox Hill Hospital

- [ ] Lifespan Inc.
- [ ] Loma Linda University Behavioral Medicine Center
- [ ] Loma Linda University Medical Center
- [ ] Long Beach Memorial Medical Center
- [ ] Long Island Jewish Medical Center
- [ ] Louis Stokes Veterans Affairs Medical Center
- [ ] Loyola University Health System
- [ ] Loyola University Medical Center
- [ ] Lucile Packard Children's Hospital (Stanford Children's Health)
- [ ] MacNeal Hospital
- [ ] Maimonides Medical Center
- [ ] Main Line Health
- [ ] Maine Medical Center
- [ ] Maricopa Medical Center
- [ ] Mary Imogene Bassett Hospital
- [ ] Massachusetts General Hospital
- [ ] Mayo Clinic Hospital Jacksonville FL
- [ ] Mayo Clinic Hospital Phoenix AZ
- [ ] Mayo Clinic Hospital Rochester MN
- [ ] Mayo Health System
- [ ] MCG Health Inc dba Augusta University Health Inc.
- [ ] McLaren Healthcare Corporation
- [ ] McLaren Regional Medical Center
- [ ] Medical Center Hospital
- [ ] Medical Center Navicent Health
- [ ] Medical University of South Carolina Medical Center
- [ ] MedStar Health

- ☐ Memorial Health Inc.
- ☐ Memorial Health System
- ☐ Memorial Health University Medical Center
- ☐ Memorial Hermann Texas Medical Center
- ☐ Memorial Hospital of Rhode Island
- ☐ Memorial Medical Center
- ☐ Memorial Sloan Kettering Cancer Center
- ☐ Mercy Hospital St. Louis
- ☐ Meridian Health System
- ☐ Methodist Healthcare University Hospital
- ☐ Methodist Hospital System
- ☐ MetroHealth Medical Center
- ☐ MetroHealth System
- ☐ Miami Children's Hospital
- ☐ Michael E. DeBakey Veterans Affairs Medical Center
- ☐ Minneapolis Veterans Affairs Medical Center
- ☐ Miriam Hospital
- ☐ Monmouth Medical Center
- ☐ Montefiore Medical Center
- ☐ Morristown Memorial Hospital
- ☐ Mount Auburn Hospital
- ☐ Mount Sinai Beth Israel
- ☐ Mount Sinai Health System
- ☐ Mount Sinai Hospital
- ☐ Mount Sinai Medical Center
- ☐ Mountain States Health Alliance
- ☐ Mt. Sinai St. Luke's/Roosevelt

- ☐ National Jewish Health
- ☐ Nationwide Children's Hospital
- ☐ Navicent Health
- ☐ New Mexico Veterans Affairs Medical Center
- ☐ New York Eye and Ear Infirmary of Mount Sinai
- ☐ New York Methodist Hospital
- ☐ New York Presbyterian Healthcare System
- ☐ New York Presbyterian Hospital
- ☐ Newark Beth Israel Medical Center
- ☐ North Shore Long Island Jewish Health System
- ☐ North Shore University Hospital
- ☐ NorthShore University Health System Evanston Hospital
- ☐ Northwestern Memorial Hospital
- ☐ Norwalk Hospital
- ☐ NYC Health and Hospitals
- ☐ NYU Hospitals Center
- ☐ NYU Langone Medical Center
- ☐ Ochsner Clinic Foundation
- ☐ Ohio State University Health System
- ☐ OhioHealth
- ☐ Oklahoma City Veterans Affairs Medical Center
- ☐ Oregon Health & Science University
- ☐ Orlando VA Medical Center
- ☐ OSF HealthCare System
- ☐ OSF Saint Francis Medical Center
- ☐ OU Medical Center
- ☐ Our Lady of The Lake Regional Medical Center

- ☐ Overlook Medical Center
- ☐ Palmetto Health
- ☐ Palmetto Health Alliance
- ☐ Parkland Health & Hospital System
- ☐ Partners HealthCare System Inc.
- ☐ Penn State Hershey Medical Center
- ☐ Philadelphia Veterans Affairs Medical Center
- ☐ Phoenix VA Health Care System
- ☐ Portland Veterans Affairs Medical Center
- ☐ Ralph H. Johnson Veterans Affairs Medical Center
- ☐ Reading Hospital and Medical Center
- ☐ Regions Hospital
- ☐ Rehabilitation Institute of Chicago
- ☐ Rhode Island Hospital
- ☐ Richard L. Roudebush Veterans Affairs Medical Center
- ☐ Robert Wood Johnson Health System
- ☐ Robert Wood Johnson University Hospital
- ☐ Roger Williams Medical Center
- ☐ Ronald Reagan UCLA Medical Center
- ☐ Roswell Park Cancer Institute
- ☐ Rush System for Health
- ☐ Rush University Medical Center
- ☐ Saint Barnabas Medical Center
- ☐ Saint Francis Care
- ☐ Saint Francis Hospital and Medical Center
- ☐ Saint Louis University Hospital
- ☐ Saint Luke's Health System

- ☐ Saint Luke's Hospital of Kansas City
- ☐ Saint Peter's University Hospital
- ☐ Saint Thomas Health
- ☐ Salem Veterans Affairs Medical Center
- ☐ San Francisco General Hospital and Medical Center
- ☐ Sanford Medical Center
- ☐ Sanford USD Medical Center
- ☐ Scott & White Hospital Temple
- ☐ Scripps Green Hospital
- ☐ Scripps Health
- ☐ Seattle Children's Hospital
- ☐ Sentara Norfolk General Hospital
- ☐ Sinai Health System
- ☐ Sinai Hospital of Baltimore
- ☐ Sinai Samaritan Medical Center
- ☐ Sioux Falls VA Health Care System
- ☐ Sioux Valley Hospitals and Health System
- ☐ Soin Medical Center
- ☐ Southeast Louisiana Veterans Health Care System
- ☐ Southern Arizona Veterans Affairs Health Care System
- ☐ Spectrum Health
- ☐ Spectrum Health Butterworth Hospital
- ☐ St. Christopher's Hospital for Children
- ☐ St. Elizabeth's Medical Center
- ☐ St. John Health System
- ☐ St. John Hospital and Medical Center
- ☐ St. Joseph Mercy Hospital

- ☐ St. Joseph's Hospital & Medical Center
- ☐ St. Louis Children's Hospital
- ☐ St. Luke's Hospital
- ☐ St. Luke's Medical Center
- ☐ St. Vincent Indianapolis Hospital
- ☐ Stanford Health Care
- ☐ Staten Island University Hospital
- ☐ Steward Health Care System
- ☐ Stony Brook University Hospital
- ☐ Strong Health System
- ☐ Strong Memorial Hospital
- ☐ Summa Akron City Hospital
- ☐ Sunnybrook Health Sciences Centre
- ☐ SUNY Downstate Medical Center/University Hospital of Brooklyn
- ☐ Swedish Medical Center
- ☐ Syracuse Veterans Affairs Medical Center
- ☐ Tampa General Hospital
- ☐ Temple University Health System
- ☐ Temple University Hospital
- ☐ Tenet Health System Hahnemann University Hospital
- ☐ Tenet Healthcare Corporation
- ☐ Texas Children's Hospital
- ☐ The Mount Sinai Hospital
- ☐ The National Institutes of Health (NIH) Clinical Center
- ☐ The Nebraska Medical Center
- ☐ The University of Texas MD Anderson Cancer Center
- ☐ Thomas Jefferson University Hospital

- ☐ Trinity Health
- ☐ Truman Medical Center Hospital Hill
- ☐ Tufts Medical Center
- ☐ Tulane Medical Center
- ☐ UAB Health System University of Alabama at Birmingham
- ☐ UC Health
- ☐ UCLA Health
- ☐ UCSD Healthcare
- ☐ UCSF Medical Center
- ☐ UF Health Jacksonville
- ☐ UF Shands Hospital
- ☐ UMass Memorial Medical Center
- ☐ UNC Health Care System
- ☐ Uniformed Services University of Health Sciences
- ☐ United Health Services Hospitals
- ☐ UnityPoint Health Des Moines
- ☐ Univ of Connecticut Health Center/John Dempsey Hospital
- ☐ Universal Health Services Inc. George Washington University Hospital
- ☐ University Health System
- ☐ University Hospital SUNY Upstate Medical University
- ☐ University Hospital, Newark, NJ
- ☐ University Hospitals and Clinics/ University of Mississippi Medical Center
- ☐ University Hospitals Case Medical Center
- ☐ University Hospitals HealthSystem
- ☐ University Medical Center New Orleans
- ☐ University of Alabama Hospital
- ☐ University of Arkansas for Medical Sciences

- ☐ University of California Davis Health System
- ☐ University of California Irvine Medical Center
- ☐ University of California San Diego Medical Center
- ☐ University of Chicago Hospitals and Health System
- ☐ University of Chicago Medical Center
- ☐ University of Cincinnati Medical Center
- ☐ University of Colorado Health
- ☐ University of Colorado Hospital
- ☐ University of Illinois at Chicago Medical Center
- ☐ University of Illinois College of Medicine at Peoria
- ☐ University of Iowa Hospitals and Clinics
- ☐ University of Kansas Hospital
- ☐ University of Kentucky Hospital
- ☐ University of Maryland Medical Center
- ☐ University of Maryland Medical System
- ☐ University of Miami Hospital
- ☐ University of Michigan Health System
- ☐ University of Michigan Medical Center
- ☐ University of Minnesota Medical Center Fairview
- ☐ University of Mississippi Medical Center
- ☐ University of Missouri Hospitals and Clinics
- ☐ University of New Mexico Hospital
- ☐ University of North Carolina Hospitals
- ☐ University of Pennsylvania Health System
- ☐ University of South Alabama Medical Center
- ☐ University of Tennessee Medical Center
- ☐ University of Texas Health Science Center at Tyler

- ☐ University of Toledo Medical Center
- ☐ University of Utah Health System
- ☐ University of Utah Hospital
- ☐ University of Vermont Medical Center
- ☐ University of Virginia Medical Center
- ☐ University of Washington Medical Center
- ☐ University of Wisconsin Hospital and Clinics
- ☐ UPMC
- ☐ UPMC Hamot
- ☐ UPMC Presbyterian Shadyside
- ☐ USC Norris Cancer Hospital
- ☐ UT Southwestern Medical Center
- ☐ UW Medicine
- ☐ VA Caribbean Healthcare System
- ☐ VA Connecticut Health Care System
- ☐ VA Nebraska Western Iowa Health Care System Omaha Division
- ☐ VA Sierra Pacific Network (10N21) Department of Veterans Affairs VISN 21
- ☐ Valley Children's Healthcare
- ☐ Vanderbilt University Medical Center
- ☐ Vanguard Health System
- ☐ VCU Medical Center
- ☐ Ventura County Medical Center
- ☐ Veterans Affairs Ann Arbor Healthcare System
- ☐ Veterans Affairs Boston Healthcare System
- ☐ Veterans Affairs Greater Los Angeles Health Care System
- ☐ Veterans Affairs Long Beach Healthcare System
- ☐ Veterans Affairs Maryland Health Care System

- [ ] Veterans Affairs Medical and Regional Office Center
- [ ] Veterans Affairs Medical Center (Atlanta)
- [ ] Veterans Affairs Medical Center James A. Haley Veterans Hospital
- [ ] Veterans Affairs Medical Center Memphis Tennessee
- [ ] Veterans Affairs New York Harbor Health Care System New York Campus
- [ ] Veterans Affairs Pittsburgh Healthcare System
- [ ] Veterans Affairs Puget Sound Health Care System
- [ ] Veterans Affairs San Diego Healthcare System
- [ ] Veterans Affairs South Texas Health Care System
- [ ] Veterans Affairs Tennessee Valley Health Care System
- [ ] Veterans Affairs Western New York Healthcare System
- [ ] Vidant Health
- [ ] Vidant Medical Center
- [ ] Wake Forest Baptist Medical Center
- [ ] Washington Hospital Center
- [ ] Wellspan Health
- [ ] Wellspan York Hospital
- [ ] Wellstar Atlanta Medical Center
- [ ] West Kendall Baptist Hospital
- [ ] West Penn Allegheny Health System
- [ ] West Virginia United Health System
- [ ] West Virginia University Hospitals Inc.
- [ ] Westchester Medical Center
- [ ] Western Pennsylvania Hospital
- [ ] Wexner Medical Center at The Ohio State University
- [ ] White Memorial Medical Center
- [ ] White River Junction VA Medical Center

- [ ] William Jennings Bryan Dorn VA Medical Center
- [ ] Winthrop South Nassau University Health System Inc.
- [ ] Winthrop University Hospital
- [ ] Women and Infants Hospital of Rhode Island
- [ ] Yale New Haven Health System
- [ ] Yale New Haven Hospital
- [ ] Other

5j. What other hospital(s) or hospital network(s) uses the PRS account "${m://ExternalDataReference}"

5k. Select the medical school that uses the PRS account "${m://ExternalDataReference}".

5l. Select the medical schools that use the PRS account "${m://ExternalDataReference}". Check all that apply.

- [ ] Albany Medical College
- [ ] Albert Einstein College of Medicine
- [ ] Baylor College of Medicine
- [ ] Boston University School of Medicine
- [ ] California Northstate University College of Medicine

- [ ] Case Western Reserve University School of Medicine
- [ ] Central Michigan University College of Medicine
- [ ] Charles E. Schmidt College of Medicine at Florida Atlantic University
- [ ] Chicago Medical School at Rosalind Franklin University of Medicine & Science
- [ ] Columbia University College of Physicians and Surgeons
- [ ] Cooper Medical School of Rowan University
- [ ] Creighton University School of Medicine
- [ ] CUNY School of Medicine
- [ ] Drexel University College of Medicine
- [ ] Duke University School of Medicine
- [ ] East Tennessee State University James H. Quillen College of Medicine
- [ ] Eastern Virginia Medical School
- [ ] Emory University School of Medicine
- [ ] Florida International University Herbert Wertheim College of Medicine
- [ ] Florida State University College of Medicine
- [ ] Frank H. Netter MD School of Medicine at Quinnipiac University
- [ ] Froedert and Medical College of Wisconsin
- [ ] Geisel School of Medicine at Dartmouth
- [ ] George Washington University School of Medicine and Health Sciences
- [ ] Georgetown University School of Medicine
- [ ] Harvard Medical School
- [ ] Hofstra Northwell School of Medicine at Hofstra University
- [ ] Howard University College of Medicine
- [ ] Icahn School of Medicine at Mount Sinai
- [ ] Indiana University School of Medicine
- [ ] Jacobs School of Medicine and Biomedical Sciences at the University at Buffalo
- [ ] Johns Hopkins University School of Medicine

- ☐ Keck School of Medicine of the University of Southern California
- ☐ Lewis Katz School of Medicine at Temple University
- ☐ Loma Linda University School of Medicine
- ☐ Louisiana State University School of Medicine in New Orleans
- ☐ Louisiana State University School of Medicine in Shreveport
- ☐ Loyola University Chicago Stritch School of Medicine
- ☐ Marshall University Joan C. Edwards School of Medicine
- ☐ Mayo Medical School
- ☐ McGovern Medical School at the University of Texas Health Science Center at Houston
- ☐ Medical College of Georgia at Augusta University
- ☐ Medical University of South Carolina College of Medicine
- ☐ Meharry Medical College
- ☐ Mercer University School of Medicine
- ☐ Michigan State University College of Human Medicine
- ☐ Morehouse School of Medicine
- ☐ New York Medical College
- ☐ New York University School of Medicine
- ☐ Northeast Ohio Medical University
- ☐ Northwestern University Feinberg School of Medicine
- ☐ Oakland University William Beaumont School of Medicine
- ☐ Ohio State University College of Medicine
- ☐ Oregon Health & Science University School of Medicine
- ☐ Pennsylvania State University College of Medicine
- ☐ Perelman School of Medicine at the University of Pennsylvania
- ☐ Ponce Health Sciences University School of Medicine
- ☐ Rush Medical College of Rush University Medical Center
- ☐ Rutgers New Jersey Medical School

- ☐ Rutgers Robert Wood Johnson Medical School
- ☐ Saint Louis University School of Medicine
- ☐ San Juan Bautista School of Medicine
- ☐ Sidney Kimmel Medical College at Thomas Jefferson University
- ☐ Southern Illinois University School of Medicine
- ☐ Stanford University School of Medicine
- ☐ State University of New York Downstate Medical Center College of Medicine
- ☐ State University of New York Upstate Medical University
- ☐ Stony Brook University School of Medicine
- ☐ Texas A&M Health Science Center College of Medicine
- ☐ Texas Tech University Health Sciences Center School of Medicine
- ☐ The Brody School of Medicine at East Carolina University
- ☐ The Commonwealth Medical College
- ☐ The University of Toledo College of Medicine
- ☐ The Warren Alpert Medical School of Brown University
- ☐ Tufts University School of Medicine
- ☐ Tulane University School of Medicine
- ☐ Uniformed Services University of the Health Sciences F. Edward Hebert School of Medicine
- ☐ Universidad Central del Caribe School of Medicine
- ☐ University of Alabama School of Medicine
- ☐ University of Arizona College of Medicine
- ☐ University of Arizona College of Medicine Phoenix
- ☐ University of Arkansas for Medical Sciences College of Medicine
- ☐ University of California Davis School of Medicine
- ☐ University of California Irvine School of Medicine
- ☐ University of California Los Angeles David Geffen School of Medicine
- ☐ University of California Riverside School of Medicine

- ☐ University of California Riverside School of Medicine
- ☐ University of California San Diego School of Medicine
- ☐ University of California San Francisco School of Medicine
- ☐ University of Central Florida College of Medicine
- ☐ University of Chicago Division of the Biological Sciences The Pritzker School of Medicine
- ☐ University of Cincinnati College of Medicine
- ☐ University of Colorado School of Medicine
- ☐ University of Connecticut School of Medicine
- ☐ University of Florida College of Medicine
- ☐ University of Hawaii John A. Burns School of Medicine
- ☐ University of Illinois College of Medicine
- ☐ University of Iowa Roy J. and Lucille A. Carver College of Medicine
- ☐ University of Kansas School of Medicine
- ☐ University of Kentucky College of Medicine
- ☐ University of Louisville School of Medicine
- ☐ University of Maryland School of Medicine
- ☐ University of Massachusetts Medical School
- ☐ University of Miami Leonard M. Miller School of Medicine
- ☐ University of Michigan Medical School
- ☐ University of Minnesota Medical School
- ☐ University of Mississippi School of Medicine
- ☐ University of Missouri Columbia School of Medicine
- ☐ University of Missouri Kansas City School of Medicine
- ☐ University of Nebraska College of Medicine
- ☐ University of Nevada School of Medicine
- ☐ University of New Mexico School of Medicine
- ☐ University of North Carolina at Chapel Hill School of Medicine

- [ ] University of North Dakota School of Medicine and Health Sciences
- [ ] University of Oklahoma College of Medicine
- [ ] University of Pittsburgh School of Medicine
- [ ] University of Puerto Rico School of Medicine
- [ ] University of Rochester School of Medicine and Dentistry
- [ ] University of South Alabama College of Medicine
- [ ] University of South Carolina School of Medicine
- [ ] University of South Carolina School of Medicine Greenville
- [ ] University of South Dakota Sanford School of Medicine
- [ ] University of Tennessee Health Science Center College of Medicine
- [ ] University of Texas at Austin Dell Medical School
- [ ] University of Texas Medical Branch School of Medicine
- [ ] University of Texas Rio Grande Valley School of Medicine
- [ ] University of Texas School of Medicine at San Antonio
- [ ] University of Texas Southwestern Medical Center Southwestern Medical School
- [ ] University of Utah School of Medicine
- [ ] University of Vermont College of Medicine
- [ ] University of Virginia School of Medicine
- [ ] University of Washington School of Medicine
- [ ] University of Wisconsin School of Medicine and Public Health
- [ ] USF Health Morsani College of Medicine
- [ ] Vanderbilt University School of Medicine
- [ ] Virginia Commonwealth University School of Medicine
- [ ] Virginia Tech Carilion School of Medicine
- [ ] Wake Forest School of Medicine of Wake Forest Baptist Medical Center
- [ ] Washington University in St. Louis School of Medicine
- [ ] Wayne State University School of Medicine

- ☐ Weill Cornell Medicine
- ☐ West Virginia University School of Medicine
- ☐ Western Michigan University Homer Stryker M.D. School of Medicine
- ☐ Wright State University Boonshoft School of Medicine
- ☐ Yale School of Medicine
- ☐ Other

5m. What other medical school(s) uses the PRS account "${m://ExternalDataReference}"?

6a. If the PRS account "${m://ExternalDataReference}" includes records associated with a Clinical and Translational Science Award (CTSA) organization, select the CTSA organization.

6b. Does the Clinical and Translational Science Award (CTSA) provide support for ClinicalTrials.gov registration and reporting?

- ○ Yes
- ○ No
- ○ Don't know

7. Does your organization use **a computer system for monitoring ClinicalTrials.gov compliance** for the PRS account "${m://ExternalDataReference}"? Answer "Yes" if your organization uses a program such as *Access*, *Excel*, *REDCap*, or *Click IRB*, to monitor problem records or to notify people about problem records.

- Yes
- No
- Don't know

7a. Approximately ***how many study records*** are currently in the PRS account for this institution (${m://ExternalDataReference})? *This number can include closed studies and studies that are in the process of being registered.*

- < 100 records
- 100 to 500 records
- 501 to 1000 records
- > 1000 records

Section 4: Policies

For clinical trials related to the PRS account "${m://ExternalDataReference}", **does your organization have a written policy**, standard operating procedure, or other form of written guidance regarding:

8. Registration on ClinicalTrials.gov?

- Yes
- No

- Don't know

9. Results reporting on ClinicalTrials.gov?

- Yes
- No
- Don't know

10a. Is your organization's registration or results reporting policy available to the public (e.g. people who are not affiliated with your organization)?

- Yes
- No
- Don't Know

10b. Please enter the website (URL) where we can find your organization's policy or policies, or describe how the public can access these policies.

For clinical trials related to the PRS account "${m://ExternalDataReference}", **does your organization have a written policy**, standard operating procedure, or other form of guidance regarding:

11. ClinicalTrials.gov records for **investigators joining** (e.g. hired by) the organization?

- Yes
- No
- Don't know

12. ClinicalTrials.gov records for **investigators leaving** the organization?

- Yes
- No
- Don't know

12a. Does your institution have a policy regarding the 2018 Common Rule Requirement for posting Informed Consent Forms? For reference: 2018 Common Rule Requirement

- Yes
- No
- Don't Know

12b. Does your institution use a quality control tool (i.e. checklist) to review registration and results submissions?

- Yes
- No
- Don't Know

Section 5: Staff

The next questions ask about functions that you and your colleagues perform related to ClinicalTrials.gov. This might include educating and

communicating with your colleagues, maintaining your PRS account, and specific tasks related to trial registration and results reporting.

13. **Including you**, how many people are employed to support ClinicalTrials.gov compliance for the PRS account "${m://ExternalDataReference}"? Count both full-time and part-time employees.

- 1 (only me)
- 2
- 3
- 4
- 5
- 6
- 7
- 8
- 9
- 10
- More than 10
- Don't know

14. When did employees of your organization begin to support investigators with ClinicalTrials.gov **registration** and **reporting** requirements for trials related to the PRS account "${m://ExternalDataReference}". Include both part- and full-time duties.

Year

Month

15a. Which of the following functions are performed by you or by other

employees of your organization? Check all that apply.

- ☐ Communicating with ClinicalTrials.gov staff (e.g. by email or telephone)
- ☐ Conducting individual (one on one) training
- ☐ Conducting workshops or group training
- ☐ Coordinating with other internal groups (i.e., IRB, grants administration, finance)
- ☐ Creating and maintaining user accounts
- ☐ Developing policies for registration and results reporting
- ☐ Enforcing or escalating compliance requirements
- ☐ Maintaining an educational website
- ☐ Notifying researchers (e.g. PIs) about sanctions for noncompliance
- ☐ Reviewing data management and/or data sharing plans
- ☐ Reviewing NIH dissemination plans
- ☐ Reviewing problem records (e.g. "Late Results per FDAAA")
- ☐ Transferring records to and from other PRS accounts
- ☐ None, this organization performs none of these functions
- ☐ Don't know

16a. Which of the following functions regarding trial **registration** are performed by you or by other employees of your organization? Check all that apply.

- ☐ Approving entries (e.g. by PIs, trial staff) into the organization's PRS account
- ☐ Assisting researchers (e.g. PIs, trial staff) with responding to PRS review comments
- ☐ Determining which studies are required to register
- ☐ Monitoring whether IRB-approved trials are registered
- ☐ Registering studies on behalf of investigators
- ☐ Responding to questions from researchers (e.g. PIs, trial staff) regarding ClinicalTrials.gov compliance (e.g. by phone or email)
- ☐ Other

☐ None, employees of this organization perform none of these functions related to trial registration

☐ Don’t know

16b. What **other** functions regarding trial **registration** are performed by you or by other employees of your organization?

17a. Which of the following **results reporting** functions are performed by you or by other employees of your organization? Check all that apply.

☐ Approving entries (e.g. by PIs, trial staff) into the organization's PRS account

☐ Assisting researchers (e.g. PIs, trial staff) with responding to PRS review comments

☐ Determining which studies are required to report results

☐ Entering results into ClinicalTrials.gov on behalf of PIs

☐ Monitoring ongoing trials for upcoming reporting requirements (e.g. to notify principal investigators and statisticians)

☐ Responding to questions from researchers (e.g. PIs) regarding ClinicalTrials.gov compliance (e.g. by phone or email)

☐ Other

☐ None, employees of this organization perform none of these functions related to results reporting

☐ Don’t know

17b. What **other results reporting** functions are performed by you or by other employees of your organization?

18. Describe your education, and describe your effort (% full-time equivalent) dedicated to ClinicalTrials.gov compliance.

	Education (check all that apply)							Effort on ClinicalTrials.gov
	PhD	MD	JD	Master's	Bachelor's	Associate's	High school	
18a. Employee 1 (me)	☐	☐	☐	☐	☐	☐	☐	

18. **Including you,** describe the people who support ClinicalTrials.gov compliance for the PRS account "${m://ExternalDataReference}". List their degree(s) and effort (% full-time equivalent) dedicated to ClinicalTrials.gov compliance.

	Education (check all the apply)							Effort on ClinicalTrials.gov
	PhD	MD	JD	Masters	Bachelor's	Associate's	High school	
18a. Employee 1 (me)	☐	☐	☐	☐	☐	☐	☐	
18b. Employee 2	☐	☐	☐	☐	☐	☐	☐	

18. **Including you,** describe the people who support ClinicalTrials.gov compliance for the PRS account "${m://ExternalDataReference}". List their

degree(s) and effort (% full-time equivalent) dedicated to ClinicalTrials.gov compliance.

	Education (check all the apply)							Effort on ClinicalTrials.gov
	PhD	MD	JD	Master's	Bachelor's	Associate's	High school	
18a. Employee 1 (me)	☐	☐	☐	☐	☐	☐	☐	
18b. Employee 2	☐	☐	☐	☐	☐	☐	☐	
18c. Employee 3	☐	☐	☐	☐	☐	☐	☐	

18. **Including you,** describe the people who support ClinicalTrials.gov compliance for the PRS account "${m://ExternalDataReference}". List their degree(s) and effort (% full-time equivalent) dedicated to ClinicalTrials.gov compliance.

	Education (check all the apply)							Effort on ClinicalTrials.gov
	PhD	MD	JD	Master's	Bachelor's	Associate's	High school	
18a. Employee 1 (me)	☐	☐	☐	☐	☐	☐	☐	
18b. Employee 2	☐	☐	☐	☐	☐	☐	☐	
18c. Employee 3	☐	☐	☐	☐	☐	☐	☐	

	Education (check all the apply)							Effort on ClinicalTrials.gov
	PhD	MD	JD	Master's	Bachelor's	Associate's	High school	
18d. Employee 4	☐	☐	☐	☐	☐	☐	☐	[dropdown]

18. **Including you,** describe the people who support ClinicalTrials.gov compliance for the PRS account "${m://ExternalDataReference}". List their degree(s) and effort (% full-time equivalent) dedicated to ClinicalTrials.gov compliance.

	Education (check all the apply)							Effort on ClinicalTrials.gov
	PhD	MD	JD	Master's	Bachelor's	Associate's	High school	
18a. Employee 1 (me)	☐	☐	☐	☐	☐	☐	☐	[dropdown]
18b. Employee 2	☐	☐	☐	☐	☐	☐	☐	[dropdown]
18c. Employee 3	☐	☐	☐	☐	☐	☐	☐	[dropdown]
18d. Employee 4	☐	☐	☐	☐	☐	☐	☐	[dropdown]
18e. Employee 5	☐	☐	☐	☐	☐	☐	☐	[dropdown]

18. **Including you,** describe the people who support ClinicalTrials.gov compliance for the PRS account "${m://ExternalDataReference}". List their

degree(s) and effort (% full-time equivalent) dedicated to ClinicalTrials.gov compliance.

	Education (check all the apply)							Effort on ClinicalTrials.gov
	PhD	MD	JD	Master's	Bachelor's	Associate's	High school	
18a. Employee 1 (me)	☐	☐	☐	☐	☐	☐	☐	▾
18b. Employee 2	☐	☐	☐	☐	☐	☐	☐	▾
18c. Employee 3	☐	☐	☐	☐	☐	☐	☐	▾
18d. Employee 4	☐	☐	☐	☐	☐	☐	☐	▾
18e. Employee 5	☐	☐	☐	☐	☐	☐	☐	▾
18f. Employee 6	☐	☐	☐	☐	☐	☐	☐	▾

18. **Including you,** describe the people who support ClinicalTrials.gov compliance for the PRS account "${m://ExternalDataReference}". List their degree(s) and effort (% full-time equivalent) dedicated to ClinicalTrials.gov compliance.

	Education (check all the apply)							Effort on ClinicalTrials.gov
	PhD	MD	JD	Master's	Bachelor's	Associate's	High school	
18a. Employee 1 (me)	☐	☐	☐	☐	☐	☐	☐	▾

	Education (check all the apply)							Effort on ClinicalTrials.gov
	PhD	MD	JD	Master's	Bachelor's	Associate's	High school	
18b. Employee 2	☐	☐	☐	☐	☐	☐	☐	
18c. Employee 3	☐	☐	☐	☐	☐	☐	☐	
18d. Employee 4	☐	☐	☐	☐	☐	☐	☐	
18e. Employee 5	☐	☐	☐	☐	☐	☐	☐	
18f. Employee 6	☐	☐	☐	☐	☐	☐	☐	
18g. Employee 7	☐	☐	☐	☐	☐	☐	☐	

18. **Including you,** describe the people who support ClinicalTrials.gov compliance for the PRS account "${m://ExternalDataReference}". List their degree(s) and effort (% full-time equivalent) dedicated to ClinicalTrials.gov compliance.

	Education (check all the apply)							Effort on ClinicalTrials.gov
	PhD	MD	JD	Master's	Bachelor's	Associate's	High school	
18a. Employee 1 (me)	☐	☐	☐	☐	☐	☐	☐	
18b. Employee	☐	☐	☐	☐	☐	☐	☐	

	Education (check all the apply)							Effort on ClinicalTrials.gov
	PhD	MD	JD	Master's	Bachelor's	Associate's	High school	
2								
18c. Employee 3	☐	☐	☐	☐	☐	☐	☐	
18d. Employee 4	☐	☐	☐	☐	☐	☐	☐	
18e. Employee 5	☐	☐	☐	☐	☐	☐	☐	
18f. Employee 6	☐	☐	☐	☐	☐	☐	☐	
18g. Employee 7	☐	☐	☐	☐	☐	☐	☐	
18h. Employee 8	☐	☐	☐	☐	☐	☐	☐	

18. **Including you,** describe the people who support ClinicalTrials.gov compliance for the PRS account "${m://ExternalDataReference}". List their degree(s) and effort (% full-time equivalent) dedicated to ClinicalTrials.gov compliance.

	Education (check all the apply)							Effort on ClinicalTrials.gov
	PhD	MD	JD	Master's	Bachelor's	Associate's	High school	
18a. Employee 1 (me)	☐	☐	☐	☐	☐	☐	☐	

	Education (check all the apply)							Effort on ClinicalTrials.gov
	PhD	MD	JD	Master's	Bachelor's	Associate's	High school	
18b. Employee 2	☐	☐	☐	☐	☐	☐	☐	
18c. Employee 3	☐	☐	☐	☐	☐	☐	☐	
18d. Employee 4	☐	☐	☐	☐	☐	☐	☐	
18e. Employee 5	☐	☐	☐	☐	☐	☐	☐	
18f. Employee 6	☐	☐	☐	☐	☐	☐	☐	
18g. Employee 7	☐	☐	☐	☐	☐	☐	☐	
18h. Employee 8	☐	☐	☐	☐	☐	☐	☐	
18i. Employee 9	☐	☐	☐	☐	☐	☐	☐	

18. **Including you,** describe the staff who support ClinicalTrials.gov compliance for the PRS account "${m://ExternalDataReference}". List their degree(s) and effort (% full-time equivalent) dedicated to ClinicalTrials.gov compliance.

	Education (check all the apply)							Effort on ClinicalTrials.gov
	PhD	MD	JD	Master's	Bachelor's	Associate's	High school	
18a. Employee 1 (me)	☐	☐	☐	☐	☐	☐	☐	
18b. Employee 2	☐	☐	☐	☐	☐	☐	☐	
18c. Employee 3	☐	☐	☐	☐	☐	☐	☐	
18d. Employee 4	☐	☐	☐	☐	☐	☐	☐	
18e. Employee 5	☐	☐	☐	☐	☐	☐	☐	
18f. Employee 6	☐	☐	☐	☐	☐	☐	☐	
18g. Employee 7	☐	☐	☐	☐	☐	☐	☐	
18h. Employee 8	☐	☐	☐	☐	☐	☐	☐	
18i. Employee 9	☐	☐	☐	☐	☐	☐	☐	
18j. Employee 10	☐	☐	☐	☐	☐	☐	☐	

18. You indicated that your organization has more than 10 employees who support ClinicalTrials.gov compliance. **Including you,** please describe the 10 people with the greatest responsibilities for supporting ClinicalTrials.gov

compliance for the PRS account "${m://ExternalDataReference}". List their degree(s) and effort (% full-time equivalent) dedicated to ClinicalTrials.gov compliance.

	Education (check all the apply)							Effort on ClinicalTrials.gov
	PhD	MD	JD	Master's	Bachelor's	Associate's	High school	
18a. Employee 1 (me)	☐	☐	☐	☐	☐	☐	☐	
18b. Employee 2	☐	☐	☐	☐	☐	☐	☐	
18c. Employee 3	☐	☐	☐	☐	☐	☐	☐	
18d. Employee 4	☐	☐	☐	☐	☐	☐	☐	
18e. Employee 5	☐	☐	☐	☐	☐	☐	☐	
18f. Employee 6	☐	☐	☐	☐	☐	☐	☐	
18g. Employee 7	☐	☐	☐	☐	☐	☐	☐	
18h. Employee 8	☐	☐	☐	☐	☐	☐	☐	
18i. Employee 9	☐	☐	☐	☐	☐	☐	☐	
18j. Employee 10	☐	☐	☐	☐	☐	☐	☐	

19a. Within the next year, does your organization **plan to hire or assign** additional staff to support ClinicalTrials.gov compliance for the PRS account "${m://ExternalDataReference}"?

- Yes
- No
- Don't know

19b. How many new people will be hired or assigned? Select the number of full time equivalents (FTEs).

20. Does your organization have a dedicated group of staff (e.g., a central office) that support ClinicalTrials.gov compliance for the PRS account "${m://ExternalDataReference}"?

- Yes
- No
- Don't know

21. Where are staff who support ClinicalTrials.gov compliance employed? If the exact name of an office or department is not included on the list below, please select the closest name. Check all that apply.

- Cancer center
- Clinical research or Clinical trials
- Corporate compliance
- Clinical and Translational Science Award (CTSA) or Clinical and Translational Science Institute (CTSI)
- Data coordinating center

- ☐ Department of biostatistics
- ☐ Departments that conduct clinical trials (e.g. Department of Psychiatry, Department of Medicine)
- ☐ Dedicated ClinicalTrials.gov office
- ☐ Institutional review board (IRB)
- ☐ Quality improvement
- ☐ Regulatory affairs
- ☐ Research administration
- ☐ Research compliance
- ☐ Research support services
- ☐ There are no staff assigned to support ClinicalTrials.gov compliance (e.g. investigators are solely responsible for compliance with these requirements)
- ☐ Don't know

Section 6: Registration

The following questions ask about trial **REGISTRATION**.

22. When did the policy about clinical trials **registration** for the PRS account "${m://ExternalDataReference}" come into effect?

Year [▾]

Month [▾]

23. Which types of clinical trials must register or are encouraged to register **according to your organization's policy**?

	Required to register	Encouraged to register, not required	Not mentioned specifically (but may be covered under the policy)	Don' know
23a. ALL investigator-initiated clinical trials (consistent with ICMJE policy)	○	○	○	○
23b. Clinical trials that include services reimbursed by Medicare or Medicaid (CMS)	○	○	○	○
23c. Clinical trials considered "applicable" under the Food and Drugs Administration Amendments Act (FDAAA)	○	○	○	○
23d. Department of Defense funded clinical trials	○	○	○	○
23e. Department of Veteran Affairs funded clinical trials	○	○	○	○
23f. NIH funded clinical trials	○	○	○	○
23g. NCI funded clinical trials	○	○	○	○
23h. PCORI funded clinical trials	○	○	○	○
23i. Trials for which the ORGANIZATION holds an IND or IDE	○	○	○	○
23j. Trials for which the INVESTIGATOR holds an IND or IDE	○	○	○	○

23. What types of clinical trials are **registered** in practice?

	Required to register	Encouraged to register, not required	Not registered	Don kno
23k. ALL investigator-initiated clinical trials (consistent with ICMJE policy)	○	○	○	○
23l. Clinical trials that include services reimbursed by Medicare or Medicaid (CMS)	○	○	○	○

	Required to register	Encouraged to register, not required	Not registered	Don knov
23m. Clinical trials considered "applicable" under the Food and Drugs Administration Amendments Act (FDAAA)	○	○	○	○
23n. Department of Defense funded clinical trials	○	○	○	○
23o. Department of Veteran Affairs funded clinical trials	○	○	○	○
23p. NIH funded clinical trials	○	○	○	○
23q. NCI funded clinical trials	○	○	○	○
23r. PCORI funded clinical trials	○	○	○	○
23s Trials for which the ORGANIZATION holds an IND or IDE	○	○	○	○
23t. Trials for which the INVESTIGATOR holds an IND or IDE	○	○	○	○

24a. Who is responsible for determining if a clinical trial **must be registered according to the policy** for the PRS account "${m://ExternalDataReference}"? Check all that apply.

- ☐ Not applicable because this responsibility is not assigned in the policy
- ☐ Institutional review board (IRB)
- ☐ Principal investigator (PI)
- ☐ PRS administrator(s)
- ☐ Other
- ☐ Don't know

24b. In practice, who is responsible for determining if a clinical trial must be **registered**? Check all that apply.

- ☐ Institutional review board (IRB)

- [ ] Principal investigator (PI) or their designee
- [ ] PRS administrator(s)
- [ ] Other
- [ ] Don't know

24b. Who else is responsible for determining if a clinical trial must be registered?

25. Does the **institutional review board (IRB) require registration** of clinical trials prior to submission or prior to initial approval?

- ○ Yes. The IRB requires registration prior to submission or approval for ALL clinical trials.
- ○ Yes. The IRB requires registration prior to submission or approval for SOME clinical trials.
- ○ No. The IRB does not require registration prior to submission or approval for any clinical trials.
- ○ Don't know

26a. When do clinical trials **have to be registered according to the policy** for the PRS account "${m://ExternalDataReference}", ?

- ○ Not applicable because this is not addressed in the policy
- ○ Before the study can be SUBMITTED to the IRB
- ○ Before the study can be APPROVED by the IRB

- Before the study BEGINS ENROLLMENT
- WITHIN 21 DAYS of starting enrollment
- The requirements differ depending on the type of trial (please explain)
- Don't know

26b. In practice, when do trials have to be **registered**?

- Not applicable. The organization does not require that trials be registered
- Before the study can be SUBMITTED to the IRB
- Before the study can be APPROVED by the IRB
- Before the study BEGINS ENROLLMENT
- WITHIN 21 DAYS of starting enrollment
- The requirements differ depending on the type of trial (please explain)
- Don't know

26c. Explain the different requirements for **registering** clinical trials of different types.

27a. Excluding enforcement by the institutional review board (IRB), does your organization enforce its **registration** policy in other ways?

- Yes
- No
- Don't Know

27b. Describe the other ways in which your organization enforces its clinical trials **registration** policy.

28a. **Who may register a clinical trial according to the policy** for the PRS account "${m://ExternalDataReference}"? Check all that apply.

- ☐ Not applicable because this responsibility is not assigned in the policy
- ☐ Institutional review board (IRB)
- ☐ Principal investigator (PI) or their designee
- ☐ PRS administrator(s)
- ☐ Other
- ☐ Don't know

28b. In practice, who may register a clinical trial on ClinicalTrials.gov? Check all that apply.

- ☐ Institutional review board (IRB)
- ☐ Principal investigator (PI) or their designee
- ☐ PRS administrator(s)
- ☐ Other
- ☐ Don't know

28c. Who else may register a clinical trial on ClinicalTrials.gov?

29a. Does the **registration** policy indicate who should be designated as the "responsible party" for trials in the PRS account "${m://ExternalDataReference}"?

- No. The policy does not indicate who should be the responsible party.
- Yes. The responsible party is the Principal Investigator or the Sponsor-Investigator.
- Yes. The responsible party is the Sponsor (e.g. the organization that received the grant).
- Yes. The responsible party is the Sponsor unless the Principal Investigator or Sponsor-Investigator holds the IND/IDE.
- Don't know

29b. In practice, who is typically designated the "responsible party" for trials in the PRS account "${m://ExternalDataReference}"?

- Principal Investigator or Sponsor-Investigator
- Sponsor (e.g. the organization that received the grant)
- Sponsor, unless the Principal Investigator or Sponsor-Investigator holds the IND/IDE

29c. Please explain why the “responsible party” role is assigned to the sponsor (e.g. the organization that received the grant).

29d. Please explain why the “responsible party” role is assigned to the principal investigator.

Section 7: Results reporting

The following questions ask about **RESULTS REPORTING**.

30. Is compliance with ClinicalTrials.gov results reporting requirements for the PRS account "${m://ExternalDataReference}" **monitored by someone other than the investigators themselves**?

- Yes
- No
- Don't know

31a. Who is **responsible for monitoring** whether clinical trial results have been reported on time **according to the policy** for the PRS account "${m://ExternalDataReference}"? Check all that apply.

- ☐ Not applicable because this responsibility is not assigned in the policy
- ☐ Institutional review board (IRB)
- ☐ Principal investigator (PI)
- ☐ PRS administrator(s)
- ☐ Other
- ☐ Don't know

31b. In practice, who is responsible for monitoring whether clinical trial results have been reported on time? Check all that apply.

- ☐ Not applicable. The organization does not monitor whether results are reported on time.
- ☐ Institutional review board (IRB)
- ☐ Principal investigator (PI) or their designee
- ☐ PRS administrator(s)
- ☐ Other
- ☐ Don't know

31c. Who else monitors compliance with **results reporting** requirements?

ClinicalTrials.gov generates a "Planning Report" for PRS Administrators.

32a. Does your organization use the Planning Report to monitor compliance with results reporting requirements?

- Yes
- No
- Don't know

32b. Does your organization use the Planning Report to notify people about results reporting deadlines **before** results are due?

- Yes
- No
- Don't know

32c. Approximately how long before results are due does your organization begin to notify investigators of the results reporting deadline?

- less than 1 month
- 1 month
- 2 months
- 3 months
- 4 months
- 5 months
- 6 months
- 7 months
- 8 months
- 9 months
- 10 months
- 11 months

- 12 months
- more than 12 months
- Don't know

33a. Which of the following software programs does your organization use to monitor ClinicalTrials.gov compliance in addition to the ClinicalTrials.gov PRS system? Check all that apply.

- We use software developed in-house.
- Access
- Excel
- Filemaker
- Forte OnCore
- Huron Research Suite
- IRBManager
- mdlogix
- ProIRB
- REDCap
- SharePoint
- Velos
- Other
- Don't know

33b. What other software programs does your organization use to monitor ClinicalTrials.gov compliance?

34. When was a computer system first used to monitor compliance for the PRS account "${m://ExternalDataReference}" with ClinicalTrials.gov registration or reporting requirements?

Year

Month

35a. Does your organization's computer system **send notifications** about records in the PRS account "${m://ExternalDataReference}" (e.g. problem records)?

- Yes
- No
- Don't know

35b. For which of the following records does your organization's computer system send notifications? Check all that apply.

- Late results per FDAAA
- Missing FDAAA information
- Never released
- Not recently updated
- PRS review comments
- Ready for review and approval
- Record has errors

- [ ] Update not released
- [ ] Don't know

36. Does your organization's computer system use an application programming interface (API) to communicate with ClinicalTrials.gov?

- ◯ Yes
- ◯ No
- ◯ Don't know

37a. Who is responsible for **entering results** according to the policy for the PRS account "${m://ExternalDataReference}"? Check all that apply.

- [ ] Not applicable because this responsibility is not assigned in the policy
- [ ] PRS administrator(s)
- [ ] Principal investigator (PI) or their designee
- [ ] Other
- [ ] Don't know

37b. In practice, who **enters results** for trials in the PRS account "${m://ExternalDataReference}"? Check all that apply.

- [ ] PRS administrator(s)
- [ ] The principal investigator (PI) or their designee
- [ ] Other
- [ ] Don't know

37c. Who else is responsible for **entering results** for completed clinical trials?

38a. Does the policy for the PRS account "${m://ExternalDataReference}" indicate that a principal investigator (PI) may be **penalized by your organization for failing to REGISTER** a trial **or for failing to POST RESULTS** on ClinicalTrials.gov?

- Yes
- No
- Don't know

38b. In practice, could a principal investigator (PI) be **penalized by your organization** for failing to **REGISTER** a trial or for failing to **POST RESULTS** on ClinicalTrials.gov?

- Yes
- No
- Don't know

38c. Which of the following penalties could be applied by your organization for failing to **REGISTER** a trial or for failing to **POST RESULTS** on ClinicalTrials.gov? Check all that apply.

- The principal investigator (PI) may not begin any new research projects
- The principal investigator (PI) may not continue with enrollment on an unregistered study
- The principal investigator (PI) may be suspended from work
- The principal investigator (PI) or department may be assessed a monetary penalty

☐ The policy indicates that the principal investigator (PI) may be penalized, but it does not describe specific penalties

☐ Other

☐ Don't know

38d. Which of the following penalties could be applied by your organization for failing to **REGISTER** a trial or for failing to **POST RESULTS** on ClinicalTrials.gov? Check all that apply.

☐ The principal investigator (PI) may not begin any new research projects

☐ The principal investigator (PI) may not continue with enrollment on an unregistered study

☐ The principal investigator (PI) may be suspended from work

☐ The principal investigator (PI) or department may be assessed a monetary penalty

☐ Other

☐ Don't know

38e. What **other** penalties could be applied by your organization for failing to register or for failing to post results?

39a. Has a principal investigator **ever been penalized** by your organization for failing to register or for failing to post results**?**

○ Yes

- ◯ No
- ◯ Don't know

39b. Please describe the circumstances under which an investigator was penalized by your organization for failing to register or for failing to post results. Do not include names or identifying information. If you prefer not to answer, you may leave this section blank.

Section 8: Consent to share responses

41a. May we share your response to this survey on a publicly accessible website ***including the name of your organization?***

We will **not** share your organization's name publicly unless you give us permission to do so.

- ◯ Yes. I agree to share the name of my organization alongside my response.
- ◯ Maybe. I would like to discuss it with the survey administrator before making a decision.
- ◯ No. You may not identify my organization.

41b. What is the best way to contact you? We will be happy to answer any questions you have before you make a decision about making your response publicly available.

42. We are identifying examples of best practices to share with other organizations. These might include educational materials for investigators (e.g. powerpoint presentations), job descriptions, checklists, manuals of procedures, spreadsheets, computer software, or anything else that relates to trial registration and results reporting. If you have any materials like these, we would be happy to help anonymize them before sharing.

Do you have any resources that you would be willing to share on the Clinical Trials Registration and Results Reporting Taskforce website (www.ctrrtaskforce.com)?

- Yes
- No

Thank you!

A member of the Clinical Trials Registration and Results Reporting Taskforce will be in contact with you to discuss these resources.

Please continue the survey - just two more questions!

Section 9: Close

42A. Was the name of your organization: ${m://ExternalDataReference} correct?

- Yes

- No

42A. Please provide the correct name of your organization:

43. How would you describe your involvement in the Clinical Trials Registration and Results Reporting Taskforce?

- Member. I call into monthly teleconferences.
- I am aware of the Taskforce, but I do not participate.
- I did not know there was a Taskforce.

44. Use the space below to describe any issues that our questions did not cover, or to provide general feedback about the survey. If there are questions you would like us to ask in our next survey, please include them here. Then click ">>" to complete the survey!

Powered by Qualtrics
